# Anaplastic thyroid carcinoma mimicking a malignant pleural mesothelioma: Clues for diagnosis

**DOI:** 10.1111/1759-7714.13191

**Published:** 2019-09-17

**Authors:** Thibaut Capron, Sophie Giusiano, Valerian Bourinet, Sophie Laroumagne, Hervé Dutau, Philippe Astoul

**Affiliations:** ^1^ Department of Thoracic Oncology, Pleural Diseases and Interventional Pulmonology North University Hospital Marseille France; ^2^ Department of Anatomic Pathology North University Hospital Marseille France; ^3^ Aix‐Marseille University Marseille France

**Keywords:** Anaplastic thyroid carcinoma, malignant pleural effusion, papillary thyroid carcinoma, pleural thyroglobulin

## Abstract

Pleural metastasis of thyroid carcinoma is very rarely encountered in the evaluation of pleural effusion and diagnosis may be challenging. However, an anaplastic transformation of papillary thyroid carcinoma (PTC), although a rare condition, should be considered even after a prolonged period of patient follow‐up. Here we report a case of anaplastic thyroid carcinoma mimicking malignant pleural mesothelioma diagnosed nine years after the initial diagnosis of PTC and detail the clues used to orient and confirm the diagnosis.

## Introduction

Metastasis of thyroid carcinoma to the pleura is a rare condition, although anaplastic transformation of previous papillary thyroid carcinoma (PTC) is one of the most aggressive cancers. In this situation, the diagnostic exploration of a prevailing pleural effusion can be a challenge for pulmonary physicians. Indeed, in usual cases of anaplastic thyroid carcinoma (ATC), metastases occur in the thyroid and regional lymph nodes rather than at a distant site. Characterization of the pleural fluid may sometimes contain subtle clues which may assist clinicians in making the correct diagnosis.

Here, we briefly report and discuss a difficult diagnostic case of anaplastic thyroid carcinoma which developed at the pleural site mimicking a malignant pleural mesothelioma.

## Case report

A 77‐year‐old patient presented with a history of progressive dyspnea of one week due to a pleural effusion on the left side. He had never smoked and retired after working as a warehouseman including a few years in shipyards where he had been exposed to asbestos. He was mainly treated for an obesity‐hypoventilation syndrome with noninvasive ventilation. He had a past medical history of PTC treated by total thyroidectomy nine years previously and additional radioiodine therapy for mediastinal relapse one year later with a prolonged survey showing he maintained a stable condition.

**Figure 1:**
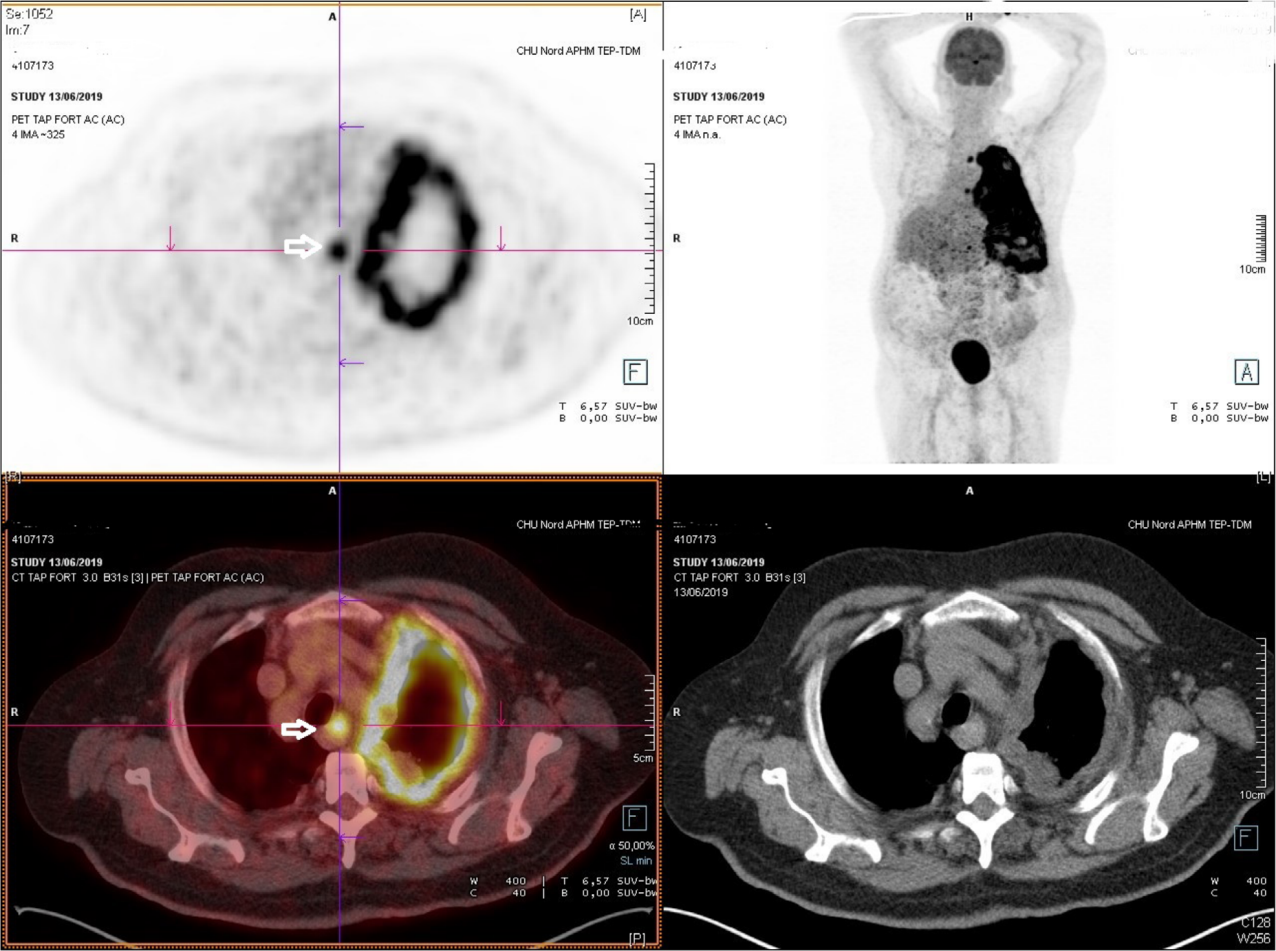
Evaluation of the patient by 18F‐fluoro‐2‐deoxy‐d‐glucose positron emission tomography/computed tomography fused imaging mimicking a malignant pleural mesothelioma with a circumferential hypermetabolic thickening of the pleura in the left pleural space. A hypermetabolic homolateral mediastinal lymph node is also visible (arrow).

On examination, the patient required 8 L/min oxygen support and chest radiograph showed a massive pleural effusion on the left side. Thoracic computed tomography (CT) detected atelectasis of the left and right lower lobes and pleural effusion on the left side. The 18F‐fluoro‐2‐deoxy‐d‐glucose positron emission tomography/computed tomography (PET/CT) fused imaging found important 18‐FDG uptake in the left pleura (SUVmax 12 g/mL,) and moderate hypermetabolism of two mediastinal (SUVmax 7.6 g/mL) and one subdiaphragmatic pillar (SUVmax 8 g/mL) nodes (Fig. 1). Because of his severe respiratory condition and despite a strong suspicion of malignant mesothelioma, a pleuroscopy for diagnosis was counterindicated. A pigtail pleural catheter was placed and percutaneous pleural biopsies performed. Analysis of the pleural fluid showed an exudate and cytology revealed predominantly lymphocytes (82%) without any malignant cells. Blood thyroglobulin was at its lowest level for the last five years (2.8 ng/mL) and thyroglobulin in the pleural fluid was at the lower detection limit (1.1 ng/mL). Echography of the neck did not detect any local relapse.

**Figure 2:**
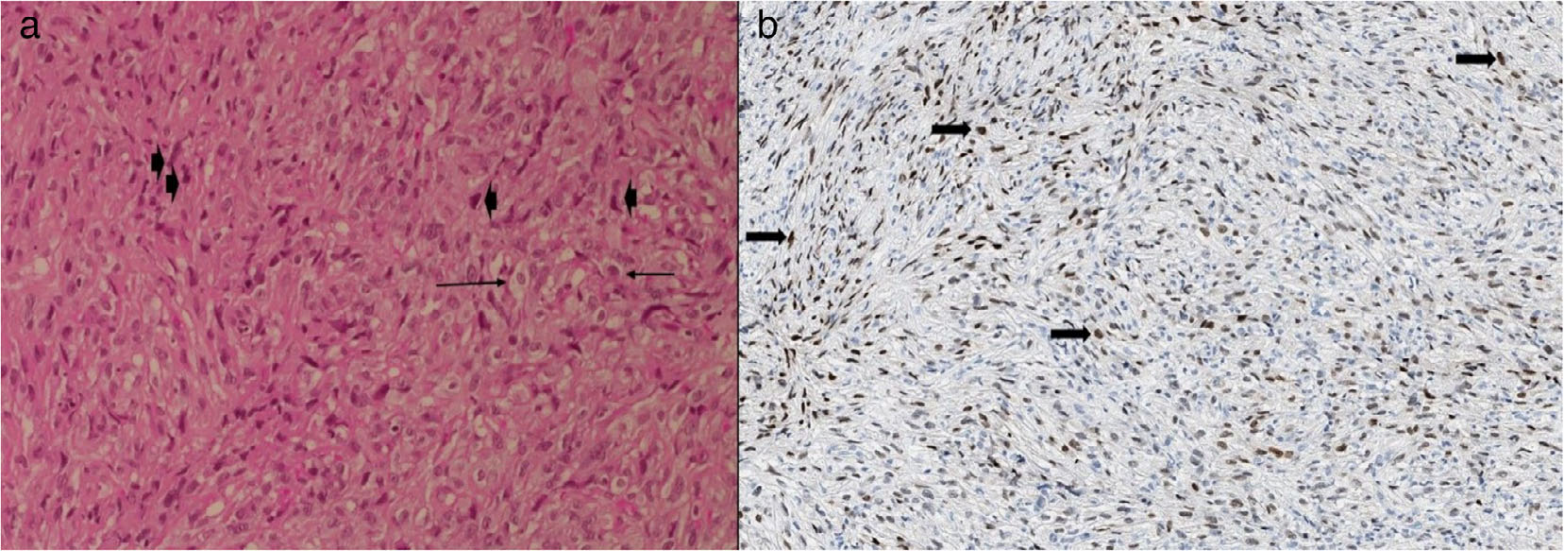
(**a**) Malignant proliferation with a storiform pattern x20. The tumor was composed of an admixture of relatively plump spindle cells with moderate atypia (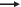) and more pleomorphic cells with high‐grade nuclei (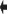). (**b**) Nuclear positivity of PAX‐8 in a large majority of tumor cells (arrows).

Pathology of the pleural biopsies showed a malignant poorly differentiated proliferation of fusiform cells (Fig. 2). Immunohistochemical staining was negative for both thyroid transcriptase factor‐1 (TTF1) and thyroglobulin, but showed strong positivity to paired‐box gene 8 (PAX‐8). Moreover, mesothelial markers such as calretinin, cytokeratin 5/6 and WT‐1 were negative. Diagnosis of anaplastic thyroid carcinoma at the pleural site was confirmed and the patient was referred to a cancer endocrinologist.

## Discussion

Thyroid cancers, the most common malignant tumors of endocrine organs, are frequently found in the papillary subtypes but rarely metastasize to the pleura. The incidence is reported to be <0.1% of malignant pleural effusions.^1^ Anaplastic transformation is a rare evolution of this disease. As a result, diagnosis of malignant pleural effusion due to anaplastic thyroid carcinoma is extremely rare and there have only been two previously published clinical cases. For Abe and colleagues a diagnosis was made following autopsy of a patient who presented with a massive pleural effusion with slowly progressive lung nodules. The lung nodules contained typical papillary thyroid carcinoma associated with undifferentiated thyroglobulin‐negative component suggestive of anaplastic transformation.^2^ Kim *et al*. reviewed fewer than 20 reported cases of anaplastic transformation at different metastatic sites and found only one case of pleural thickening with pleural effusion in which diagnosis was made by pleural biopsies with immunohistochemical staining suggestive of both PTC and anaplastic components.^3^

In our case, the clinical work‐up was led by the diagnosis of a pleural exudate in an asbestos‐exposed patient with a PET‐CT evaluation mimicking a malignant pleural mesothelioma. It highlights the diagnostic difficulties in such situations of malignant pleural effusion in a patient with a previous history of PTC, in particular when a thoracoscopic assessment of the pleural cavity is not feasible.

First, a simple dosage of thyroglobulin in the pleural effusion may be helpful. This protein is known as a marker of PTC relapse after total thyroidectomy when the serum level is high, reflecting the presence of thyroid papillary cells. Following this suggestion, it has been reported that elevated pleural fluid thyroglobulin could be a potential marker of pleural metastasis from PTC.^4^ However, ATC is known for losing thyroglobulin or TTF1 expression.^5^ Unlike the two previously reported cases, our case did not show mixed cellular proliferation, only ATC histology. This is in accordance with the low level of pleural fluid thyroglobulin found in our patient.

Radioiodine‐based imaging is not helpful in the case of ATC as undifferentiated cells do not absorb the iodine, but PET‐CT provided important metabolic information.

A final diagnosis was obtained from tissue morphology and immunohistologic profile in all the cases discussed here. ATC is typically negative for thyglobulin and TTF1, and the PAX‐8 transcription factor appears to have a useful diagnostic value (expressed in 79% of ATC cells).^1^, ^6^ This marker, a nephric‐lineage transcriptor factor for organogenesis of the thyroid gland, kidney, and mullerian system, is mainly shared by renal carcinoma. BRAF mutation that was found in our patient may also help since it is commonly found in thyroid carcinoma, but not in primary pleural processes such as mesothelioma. Similar disease‐specific markers may also be useful to differentiate other rare tumors or diseases developing in the pleura, such as multiple myeloma.^7^

In conclusion, we report the first case of single‐component anaplastic thyroid carcinoma at the unique pleural metastatic site mimicking a malignant pleural mesothelioma. Owing to its scarcity and poor prognosis, this disease may be underdiagnosed. It could be evoked typically in patients with a past history of PTC, even after long‐term patient follow‐up. Pleural thyroglobulin, PAX‐8 immunostaining and molecular analysis may also be useful to orient and confirm the diagnosis.

## Disclosure

No authors report any conflict of interest.

## References

[1] Vyas M , Harigopal M . Metastatic thyroid carcinoma presenting as malignant pleural effusion: A cytologic review of 5 cases. Diagn Cytopathol 2016; 44: 1085–9. 27456348 10.1002/dc.23547

[2] Abe T , Suzuki M , Shimizu K *et al* Anaplastic transformation of papillary thyroid carcinoma in multiple lung metastases presenting with a malignant pleural effusion: A case report. J Med Case Reports 2014; 8: 460. 10.1186/1752-1947-8-460 PMC4307622 25532447

[3] Kim H , Park YW , Oh Y‐H , Sim J , Ro JY , Pyo JY . Anaplastic transformation of papillary thyroid carcinoma only seen in pleural metastasis: A case report with review of the literature. Head Neck Pathol 2017; 11: 162–7. 27550513 10.1007/s12105-016-0751-4 PMC5429274

[4] Rosenstengel A , Lim EM , Millward M , Lee YG . A distinctive colour associated with high iodine content in malignant pleural effusion from metastatic papillary thyroid cancer: A case report. J Med Case Reports 2013; 7: 147. 10.1186/1752-1947-7-147 PMC3680221 23724969

[5] Olson MT , Nuransoy A , Ali SZ . Malignant pleural effusion resulting from metastasis of thyroid primaries: A cytomorphological analysis. Acta Cytol 2013; 57: 177–83. 23406984 10.1159/000345696

[6] Bishop JA , Sharma R , Westra WH . PAX8 immunostaining of anaplastic thyroid carcinoma: A reliable means of discerning thyroid origin for undifferentiated tumors of the head and neck. Hum Pathol 2011; 42: 1873–7. 21663937 10.1016/j.humpath.2011.02.004

[7] Wang Z , Xia G , Lan L *et al* Pleural effusion in multiple myeloma. Intern Med 2016; 55: 339–45. 26875957 10.2169/internalmedicine.55.4733

